# Effect of platelet-rich plasma on peri-implant bone repair

**DOI:** 10.6026/973206300212845

**Published:** 2025-08-31

**Authors:** Mohammad Jalaluddin, Rohit Sharma, Deepika Yadav, Anish Ashok Gupta, Ruchi Raval, Muzammil Moin Ahmed, Tanvi Hirani

**Affiliations:** 1 Department of Periodontics and Implantology, Kalinga Institute of Dental Sciences, KIIT Deemed University, Bhubaneswar-751024, India; 2 Department of Dentistry, Amaltas institute of medical sciences, Dewas, Madhya Pradesh, India; 3 Consultant Endodontist, Captain Nandlal Yadav Multi-Speciality Hospital, Rewari, Haryana, India; 4 Department of Oral Pathology & Microbiology, People's Dental Academy, People's University, Bhopal, Madhya Pradesh, India; 5 Department of Periodontics and Implantology, K M Shah Dental College and Hospital, Sumandeep Vidyapeeth Deemed to be University, Vadodara, Gujarat, India; 6 Department of Public Health, College of Applied Medical Sciences, Qassim University, Buraydah 51452 P.O. Box 6666, Saudi Arabia; 7 Department of Periodontology and Implantology, Karnavati University, Gujarat, India

**Keywords:** Platelet-rich plasma, peri-implant bone, osseointegration, dental implants, bone regeneration, CBCT

## Abstract

Osseointegration and peri-implant bone regeneration are critical determinants of long-term dental implant success, yet achieving optimal outcomes remains challenging. Platelet-Rich Plasma (PRP), enriched with autologous growth factors, has been proposed as an adjunctive biomaterial to enhance bone healing. In this randomized controlled clinical trial, 30 patients requiring single implants were allocated to PRP-coated (Group A) and non-PRP (Group B) groups. Group A demonstrated significantly higher implant stability quotient (ISQ) values and peri-implant bone density compared to controls at 3 months. Thus, we show that PRP application may effectively improve implant stability and peri-implant bone regeneration.

## Background:

The success of dental implant therapy largely depends on effective osseointegration and the quality of surrounding bone [1]. In recent years, regenerative techniques have gained prominence in enhancing peri-implant bone healing, among which Platelet-Rich Plasma (PRP) has emerged as a potential adjunctive biomaterial [2]. PRP is an autologous concentration of platelets in a small volume of plasma, containing a rich source of growth factors such as platelet-derived growth factor (PDGF), transforming growth factor-beta (TGF-β) and vascular endothelial growth factor (VEGF) that stimulate tissue regeneration and healing [3]. The rationale behind using PRP in implant dentistry stems from its ability to accelerate early bone formation and enhance vascularization at the implant site [4]. Several preclinical and clinical studies have demonstrated that PRP can improve the bone-to-implant contact (BIC) and increase bone density around implants, especially in compromised bone conditions [5, 6]. Its ease of preparation, autologous origin and bioactive properties make it a minimally invasive and safe approach to promote osseointegration [7]. Despite the promising biological potential of PRP, evidence regarding its clinical efficacy in peri-implant bone regeneration remains inconclusive. Some studies have shown significant improvements in implant stability and peri-implant bone levels, while others report minimal or no added benefits [8, 9]. The variability in PRP preparation protocols, patient factors and implant site characteristics may contribute to these discrepancies [10]. In clinical dentistry, especially in implantology, achieving and maintaining adequate bone volume around the implant is critical to ensure long-term success [11]. The early stages of healing around an implant involve a cascade of biological processes, including inflammation, proliferation and tissue remodeling [12]. PRP contributes positively during these phases by releasing growth factors that attract osteoblasts and mesenchymal stem cells to the defect site, thereby promoting bone matrix formation [13]. Moreover, PRP enhances angiogenesis, which is essential for delivering oxygen and nutrients required for tissue regeneration [14]. Therefore, it is of interest to report the clinical effectiveness of PRP in enhancing peri-implant bone healing and osseointegration through radiographic and clinical assessments.

## Materials and Methods:

A total of 30 partially edentulous patients, aged between 25 and 55 years, were enrolled based on inclusion and exclusion criteria. Ethical approval was obtained from the institutional ethical committee and written informed consent was secured from all participants.

## Inclusion criteria:

[1] Patients requiring single-tooth replacement with a dental implant in the mandibular posterior region

[2] Good general health and oral hygiene

[3] Adequate bone volume without the need for grafting

## Exclusion criteria:

[1] Smokers and tobacco users

[2] Patients with systemic conditions affecting bone healing (e.g., diabetes, osteoporosis)

[3] History of periodontal disease or bisphosphonate therapy

[4] Pregnant or lactating women

The participants were randomly divided into two groups:

[1] Group A (PRP Group): Received dental implants with Platelet-Rich Plasma application

[2] Group B (Control Group): Received dental implants without PRP

## Preparation of PRP:

Approximately 10 mL of venous blood was collected from patients in Group A using sterile vacutainers containing anticoagulant. The blood was subjected to a two-step centrifugation process: the first spin at 1500 rpm for 10 minutes to separate the plasma, followed by a second spin at 3000 rpm for 10 minutes to concentrate the platelets. The PRP layer was collected and activated using calcium chloride prior to application.

## Surgical procedure:

All implants were placed under local anesthesia following standard surgical protocols. In Group A, the PRP was applied to the osteotomy site and implant surface prior to placement. In Group B, implants were placed without PRP. Healing abutments were placed and patients were advised standard post-operative care.

## Assessment parameters:

[1] Implant stability: Measured using Resonance Frequency Analysis (RFA) to obtain Implant Stability Quotient (ISQ) values at baseline (immediately after placement), 1 month and 3 months.

[2] Bone density: Evaluated using Cone Beam Computed Tomography (CBCT) at baseline and at 3 months to assess changes in peri-implant bone density.

## Statistical analysis:

Data were compiled and analyzed using SPSS software version 25.0. Paired t-tests and one-way ANOVA were used to compare intergroup and intragroup differences over time. A p-value < 0.05 was considered statistically significant.

## Results:

All 30 participants completed the study without any reported postoperative complications or implant failures. The clinical and radiographic parameters were evaluated at baseline, 1 month and 3 months. Group A (PRP group) showed a consistent increase in ISQ values over time. At baseline, the mean ISQ was 63.8 ± 2.1, increasing to 68.2 ± 1.9 at 1 month and 72.4 ± 2.3 at 3 months. In contrast, Group B (control) demonstrated lower mean ISQ values at the same intervals: 62.9 ± 2.5 at baseline, 65.4 ± 2.3 at 1 month and 68.1 ± 3.1 at 3 months. The difference between the two groups was statistically significant at 3 months (p < 0.05) (Table 1 - see PDF). CBCT measurements showed increased bone density in both groups, with Group A exhibiting higher values at 3 months. Group A improved from 726 ± 45 HU at baseline to 856 ± 42 HU at 3 months, while Group B improved from 718 ± 41 HU to 768 ± 38 HU. The intergroup difference at 3 months was statistically significant (Table 2 - see PDF). The mean gain in implant stability from baseline to 3 months was 8.6 ± 2.2 in Group A and 5.2 ± 2.7 in Group B. The gain was significantly higher in the PRP group (Table 3 - see PDF). These findings suggest that PRP enhances both implant stability and peri-implant bone regeneration significantly over a 3-month period.

## Discussion:

The present study evaluated the clinical and radiographic effectiveness of Platelet-Rich Plasma (PRP) in enhancing peri-implant bone regeneration and implant stability. Our findings demonstrated significantly improved implant stability quotient (ISQ) values and greater peri-implant bone density in the PRP group compared to the control group. These outcomes are consistent with previous reports supporting the regenerative potential of PRP in implant dentistry [1, 2]. PRP is known to contain a high concentration of autologous growth factors such as PDGF, TGF-β, IGF and VEGF, which contribute to cell proliferation, chemotaxis, angiogenesis and extracellular matrix formation [3, 4]. These biological actions may explain the improved early osseointegration observed in our PRP group. Similar enhancements in ISQ values with PRP have been documented by Choi *et al.* who reported superior primary stability and earlier functional loading capability in implants treated with PRP [5]. The observed increase in bone density as measured by CBCT further supports the role of PRP in enhancing mineralization around implants. Bone healing is accelerated when angiogenesis and osteoblastic activity are enhanced, both of which are facilitated by growth factors released from activated PRP [6, 7]. This correlates with the findings of Gürbüzer *et al.* who reported greater bone-to-implant contact (BIC) and improved peri-implant bone density with PRP use in animal models [8]. However, despite promising results, some studies have reported inconsistent or limited benefits from PRP application. A systematic review by Del Fabbro *et al.* found that while PRP can improve early healing, its long-term effects on implant survival and marginal bone loss remain inconclusive [9]. Such discrepancies may be attributed to differences in PRP preparation techniques, platelet concentration, presence of leukocytes and activation methods [10, 11]. Moreover, the clinical outcome of PRP may be influenced by patient-specific factors such as systemic health, bone quality and oral hygiene status. In our study, careful patient selection and standardization of PRP preparation helped reduce such confounding variables. Nonetheless, it is important to recognize that PRP is not a substitute for meticulous surgical technique and proper implant placement, which remain critical to implant success [12, 13]. Limitations of our study include a relatively short follow-up period and a small sample size. Although significant differences were noted at 3 months, longer-term studies are necessary to assess whether these early gains translate into improved long-term implant survival and stability. Additionally, advanced imaging techniques such as micro-CT or histomorphometry may offer more detailed insights into bone remodeling patterns and quality of osseointegration [14, 15]. Platelet-rich plasma (PRP) is widely explored in implant dentistry for its potential to stimulate bone repair, but its effectiveness in consistently enhancing peri-implant healing remains uncertain [16].

## Conclusion:

The use of PRP as an adjunctive biomaterial for promoting peri-implant bone healing is shown. Future research should aim to establish standardized protocols for PRP preparation and application and explore its synergistic potential when combined with bone grafts or biomimetic coatings.
